# Beyond Wrinkle Efficacy: Toward a Broader Assessment of Longitudinal Compatibility in Routine Upper-Face Aesthetic BoNT-A

**DOI:** 10.3390/toxins18050232

**Published:** 2026-05-19

**Authors:** Andrea Felice Armenti

**Affiliations:** LA VISION Training Institute, Via Magenta, 5, 00199 Rome, Italy; andrea.armenti@lavisiontin.com

**Keywords:** botulinum toxin A, esthetic medicine, upper face, longitudinal assessment, facial expressivity, muscle adaptation, wrinkle efficacy, treatment compatibility

## Abstract

Botulinum toxin A (BoNT-A) is well established for the esthetic treatment of upper-face dynamic lines, and long-term adult studies suggest that repeated treatment may remain effective and acceptable over time in many patients. However, wrinkle efficacy and patient satisfaction do not by themselves determine whether repeated treatment remains acceptable in broader morphologic, dynamic–expressive, and longitudinal terms. At the same time, the toxin’s pharmacology entails finite presynaptic blockade followed by active synaptic and architectural recovery, and imaging, histological, and neurophysiological studies indicate that repeated chemodenervation cannot be assumed to be biologically neutral at the muscle level. The resulting problem is not whether BoNT-A works, but whether current outcome frameworks are sufficient to judge repeated-treatment compatibility in full. Here, structural tolerance is proposed as a provisional clinical lens for an underdescribed boundary: whether repeated-treatment compatibility is fully captured by wrinkle reduction and patient acceptability alone. The paper organizes this problem across morphologic, dynamic–expressive, and longitudinal domains, outlines candidate warning signs, and develops a pragmatic tiered approach to assessment spanning routine care, structured clinical follow-up, and research-oriented evaluation. The aim is to support more complete longitudinal thinking in upper-face aesthetic BoNT-A, to clarify what current outcome frameworks remain unaddressed, and to identify priorities for future empirical study.

## 1. Introduction

Botulinum toxin A (BoNT-A) is well established in the esthetic treatment of the upper face. Its efficacy in reducing dynamic lines in the forehead, glabella, and lateral canthal region is not in question, and long-term adult studies suggest that repeated treatment may remain effective and acceptable over time in many patients without obvious loss of clinical utility [1,2,3,4]. These findings do not define long-term compatibility by themselves.

Botulinum toxin A acts presynaptically at the neuromuscular junction by cleaving SNAP-25 and blocking acetylcholine release, producing a chemical denervation of the targeted muscle fibers without direct structural lesion of the fiber itself [5,6]. The clinical effect on upper-face dynamic lines is finite: in adult esthetic practice, peak action is typically reached within 1–2 weeks and apparent clinical duration is in the order of three to four months, after which axonal sprouting and synaptic remodeling progressively restore neuromuscular transmission [6,7,8]. Two consequences follow. First, treatment is necessarily cyclical: durable esthetic benefit in the upper face is achieved through repeated exposure, not through a single intervention. Second, recovery between cycles is biologically active rather than passive; the substrate that receives the next dose is the substrate that has been remodeled by the previous one. The repeated-treatment compatibility question that motivates this Perspective is therefore not separable from the toxin’s pharmacology: it is precisely because chemodenervation is reversible but recovery is reorganizational that cumulative exposure across cycles cannot, on pharmacological grounds alone, be presumed to leave the muscular and peri-muscular substrate identical to baseline.

Current discussions in esthetic BoNT-A often remain divided between two partial perspectives. One emphasizes durable wrinkle benefit and sustained patient satisfaction [1,2]. The other shows that facial muscle is not a biologically neutral target, as imaging-based, histological, and neurophysiological studies have documented treatment-related muscle adaptation in different contexts [5,6,7,8]. Repeated chemodenervation may be associated with atrophy, altered muscle architecture, or incomplete functional recovery in some settings [5,6,7,8,9], and localized post-treatment muscle changes have also been reported in facial muscles [8]. Together, these studies show that wrinkle efficacy can remain durable while repeated treatment cannot automatically be assumed to be biologically neutral. What remains less clear is how to judge the compatibility between these two observations during repeated upper-face treatment.

This question is especially relevant on the upper face. The forehead, glabellar complex, and periocular region are not only wrinkle-bearing esthetic units. They also contribute to emotional signaling, nonverbal communication, and socially meaningful modulation of facial expression [10,11,12]. In addition, emerging anatomical perspectives suggest that upper-face treatment should not always be understood as isolated weakening of single wrinkle-generating muscles, but in relation to interconnected regional dynamics [13]. Therefore, a treatment may remain successful in conventional wrinkle terms while still introducing subtler changes in dynamic range, expressive naturalness, or regional morphologic coherence. These dimensions are not necessarily captured by a wrinkle-centered assessment alone [10,11,14,15].

Additional caution comes from outside esthetic practice, but the inference must be drawn with care. In the pediatric spasticity literature, repeated exposure to BoNT-A has been associated with persistent or slowly recovering changes in muscle morphology and growth trajectories [16,17,18,19]. The role of this literature in the present Perspective is strictly delimited. It is invoked here only to establish a single, narrow point: that BoNT-A exposure is capable of producing muscle-level changes that do not fully reverse between cycles under conditions of repeated cumulative dosing. It is not invoked as a clinical analog of routine adult upper-face esthetic practice, and the present manuscript does not claim that adult esthetic exposure produces outcomes comparable in magnitude, distribution, or clinical relevance to those observed in pediatric spasticity. The disqualifying differences are explicit: pediatric spasticity involves pathologic muscle under active spastic drive, cumulative doses substantially higher than those used in upper-face esthetics, a developing musculoskeletal system with concurrent growth and remodeling, and a therapeutic rather than esthetic intent. None of these conditions applies to routine adult facial treatment. The legitimately translatable element is therefore biological plausibility alone—the working position that the adult esthetic upper face cannot be presumed a priori to be biologically inert over repeated cycles—and on this basis alone the pediatric data justify a compatibility-oriented framework for adult esthetic follow-up.

The central problem is not efficacy itself, but whether current esthetic outcome frameworks are sufficient to judge repeated upper-face treatment in broader morphologic, dynamic–expressive, and longitudinal terms. As summarized in Table 1, the literature addresses several components of this problem but does not yet provide an integrated way to evaluate the compatibility between them.

This paper introduces the term structural tolerance as a provisional clinical lens for this underdefined assessment boundary. The term is used to name a gap between what current studies capture well—such as wrinkle efficacy and patient satisfaction—and what they may undercapture, such as cumulative muscular adaptation, dynamic–expressive change, and greater longitudinal compatibility. The contribution is one of problem formulation and preliminary operational framing rather than construct validation. Consistent with the Perspective format of Toxins, the manuscript is structured as an argument-driven proposal rather than as a systematic synthesis of primary efficacy data: it identifies an unresolved problem at the boundary between esthetic outcome and toxin-driven biological adaptation, and articulates how it could be approached clinically and empirically.

## 2. A Provisional Clinical Lens for Longitudinal Compatibility

The esthetic literature on upper-face BoNT-A supports long-lasting wrinkle reduction, while biologic and imaging studies suggest that repeated exposure may not be completely neutral at the muscle level [1,2,3,4,5,6,7,8]. The unresolved question is how repeated-treatment compatibility should be judged when both observations are true.

In this paper, structural tolerance denotes a clinical judgment on whether a repeatedly treated upper-face region maintains an acceptable balance between wrinkle benefit and broader regional compatibility over time. The term is used clinically, rather than histologically, to frame a question that current outcome frameworks do not fully resolve: whether maintained wrinkle improvement remains compatible with preserved regional form, usable facial dynamics, and acceptable expressive function across repeated cycles [1,5,10,14]. The term is used in a morphological and functional sense and should not be confused with immunological tolerance or antibody-mediated resistance to BoNT-A, which are distinct phenomena addressed elsewhere in the literature.

Conventional endpoints do not fully capture this problem. Wrinkle severity scales may continue to show benefit, and patient satisfaction may remain high, without excluding progressive narrowing of expressive range, reduced modulation in facial tasks, or gradual loss of regional morphologic coherence [10,11,14]. In contrast, the biologic plausibility of muscle adaptation does not establish clinically relevant incompatibility in routine esthetic care [5,6,7,8]. The practical gap, therefore, is how to approach this broader compatibility boundary clinically.

This broader frame is especially relevant on the upper face, where esthetic outcome cannot be fully separated from the functional display and where the visible benefit may not be perfectly aligned with the biologic adaptation over time [8,10,11,12,16,18,19,20]. A treatment may therefore remain technically effective while becoming less compatible in broader regional or expressive terms. The clinical question is whether the benefit achieved by the wrinkle remains proportional to its cumulative regional cost.

At a mechanistic level, three complementary and testable hypotheses can be advanced to link chemodenervation to broader peri-muscular remodeling, and they are offered here as candidate routes for empirical work rather than as established pathways. First, repeated chemodenervation can produce selective fiber-type atrophy and reduced cross-sectional area within the treated muscle, with preferential involvement of type II fibers and partial substitution of fibro-adipose tissue, consistent with imaging and histological evidence in injection contexts where this has been examined [5,7,8,9]. Second, sustained reduction of contractile activity at the muscle–reticular dermis interface is hypothesized to reduce the mechanical conditioning normally transmitted to the overlying soft-tissue envelope; over repeated cycles, this may contribute to progressive thinning of regional soft-tissue support and to the morphologic cues described under morphological tolerance (periocular hollowing tendency, brow flattening, and regional skeletonization). Third, denervated and chronically under-loaded muscle exhibits altered myokine and secretome output (involving the myostatin, IL-6, and IGF-1 axes), and a paracrine influence on adjacent dermal fibroblast and adipocyte behavior is biologically plausible. None of these mechanisms have been demonstrated specifically in the adult upper-face esthetic context, and the present manuscript does not claim otherwise. They are advanced as falsifiable hypotheses that the structural-tolerance lens makes available for future imaging-, histology-, and biomarker-informed studies [5,6,7,8,18,19].

The sections that follow organize this lens into three domains—morphologic, dynamic–expressive, and longitudinal—and then translate it into a tiered approach to assessment for routine injectors, structured clinical settings, and research-oriented evaluation.

## 3. Domains and Candidate Warning Signs Relevant to Longitudinal Compatibility

For clinical use, this lens can be organized into three domains for serial follow-up: morphologic, dynamic–expressive, and longitudinal tolerance. The aim is not to identify a single adverse feature, but to judge whether the treated region remains acceptable across dimensions that wrinkle improvement alone may not capture. These domains are analytic rather than validated and are intended to structure serial observation and future study design.

Table 2 maps how the three domains are hypothesized to interact within the structural-tolerance lens, together with the upstream input (repeated BoNT-A exposure) and the downstream slice captured by conventional endpoints (wrinkle benefit and patient satisfaction).

### 3.1. Morphologic Tolerance

Morphologic tolerance refers to the apparent preservation of regional form and architectural coherence during repeated treatment cycles. Clinically, it concerns whether the treated upper-face region continues to appear balanced and structurally congruent, rather than progressively flattened, hollowed, over-deflated, or visually depleted. In routine practice, changes in volume impression, contour harmony, or brow–periocular balance are aspects that wrinkle severity scales alone do not capture [5,6,8,20].

### 3.2. Dynamic–Expressive Tolerance

Dynamic–expressive tolerance refers to the apparent preservation of usable facial movement and expressive modulation. This domain is especially relevant on the upper face, where the forehead, the glabellar complex, and the periocular region contribute to nonverbal communication, emotional signaling, and task-dependent facial variation [10,11,12,15]. A treatment may reduce dynamic lines while also narrowing expressive range, reducing brow mobility beyond what is esthetically necessary, or reducing periocular participation during smiling. These changes may not produce immediate dissatisfaction, but may still suggest reduced expressive compatibility [10,11,14].

### 3.3. Longitudinal Tolerance

Longitudinal tolerance refers to the sustainability of the wrinkle benefit–naturalness–expressivity balance across time. It becomes relevant when outcomes remain acceptable after a single cycle but appear progressively less forgiving after repeated treatment. Clinically, this may be reflected by a narrowing of the acceptable treatment window, a growing need for caution to avoid flatness or rigidity, a gradual decoupling between wrinkle improvement and perceived naturalness, or increasing reliance on protocol adjustment across cycles. Candidate signs include shorter tolerated intervals, more frequent corrective refinement, or reduced room for dose flexibility, even when the visible wrinkle benefit remains satisfactory. This domain concerns a trajectory rather than a snapshot: whether repeated treatment remains proportionate over time, not simply whether it continues to work in the short term [1,2,5,6].

These domains are analytically distinct but clinically interconnected, and the relevant signal lies in the pattern that emerges when regional form, functional movement, and serial compatibility are considered together. Table 3 summarizes the three domains and representative warning signs. An expanded candidate list of warning signs is provided in the Supplementary Table S1.

## 4. A Pragmatic Tiered Approach to Assessment

The assessment should remain proportionate to the setting, expertise, and purpose. Not every injector can be expected to perform a research-grade evaluation, but the wrinkle response alone is unlikely to capture the full range of outcomes that may matter over repeated cycles. A tiered scaffold is therefore more realistic than a single universal standard, allowing the problem to be approached through routine clinical proxies, more structured comparative follow-up, and research-level compatibility markers. The model is intentionally illustrative rather than prescriptive: the aim is not to define a validated clinical protocol but to suggest how existing routine practice might already incorporate a broader compatibility lens without waiting for formal validation. Proposing a more detailed or operationalized framework at this stage would risk overstating the current evidence base and misrepresenting the construct as established rather than provisional [1,5,10,11].

### 4.1. Level 1: Routine Clinical Assessment

At the first level, structural tolerance is approached through a basic assessment feasible in routine practice. The aim is not to diagnose remodeling or quantify subtle morphologic changes with precision but to avoid judging repeated upper-face treatment solely on wrinkle improvement. At minimum, this level should include standardized photographs or short video documentation in repose and during simple facial tasks, including brow elevation, frown, and, when the periocular region is treated, smiling. The clinician can then assess wrinkle benefit together with brow balance, regional naturalness, dynamic range, and visible warning signs throughout the cycles. Existing consensus recommendations for upper-face documentation provide a practical reference for this minimum standard [10,11,14,21,22].

### 4.2. Level 2: Structured Clinical Assessment

The second level is intended for structured clinical settings, teaching environments, or centers with a stronger follow-up culture. Here, assessment becomes more comparative and less dependent on impression alone. Serial comparison across treatment cycles becomes more important and third-observer evaluation may be added to reduce single-clinician bias. More structured clinical scoring can be introduced and simple ultrasound or other pragmatic adjuncts may be considered when available. Structured dynamic–expressive scoring may also be used when available [12,23,24].

### 4.3. Level 3: Research-Grade Assessment

The third level is research-grade assessment. Its purpose is not routine decision making, but empirical exploration of conditions under which compatibility may become less clear. This level is best served by repeated-cycle longitudinal designs, more homogeneous cohorts, better-defined exposure patterns, and integrated endpoints that include morphological, expressive, and wrinkle outcomes. Observer-layered assessment is likely to be important because subtle expressive changes may not be adequately captured by patient satisfaction alone. Imaging-informed approaches, including advanced ultrasound-based measures or other morphologic assessment techniques, may help distinguish visible esthetic benefit from less obvious morphologic adaptation. At this level, the key question is no longer simply whether treatment works in practice, but under what conditions repeated treatment remains compatible between cycles, regions, and patient profiles [5,6,7,14,18,19,20,24].

A tiered model also helps prevent overinterpretation. Level 1 can identify clinical warning signs, but it cannot establish causality or characterize tissue-level mechanisms. Level 2 can strengthen longitudinal judgment and expressive assessment, but it remains partly dependent on local protocols and observer quality. Level 3 can investigate the compatibility boundary more rigorously, but its conclusions may not be immediately generalizable to all routine esthetic settings. Table 4 summarizes this logic. An illustrative list of tier-specific assessment components is provided in the Supplementary Table S2.

## 5. Clinical Questions and Considered Responses

The framework proposed here raises questions that clinicians and researchers may find difficult to answer with currently available evidence. Table 5 addresses seven questions that are frequently implicit in discussions of long-term upper-face BoNT-A, and for which the existing literature supports a considered—if necessarily qualified—response. The answers are not definitive; they reflect the current state of evidence and are intended to make explicit what can and cannot be concluded at this stage.

## 6. Clinical Implications, Current Limits, and Research Priorities

### 6.1. Clinical Implications

The main clinical implication is not to displace wrinkle efficacy, but to avoid treating it as the sole endpoint of repeated BoNT-A in the upper face. The broader question of how localized interventions should be interpreted when their intended results are global, adaptive, and longitudinal has been addressed elsewhere in the context of neuromuscular medicine [25]. The present paper applies a compatible logic specifically to the repeated-treatment compatibility problem in upper-face esthetic BoNT-A. In many patients, repeated treatment remains effective and acceptable over time [1,2]; the additional question is whether regional form, expressive usability, and longitudinal naturalness remain proportionately preserved throughout the cycles [5,6,10,11,12,14].

### 6.2. Current Limits of the Evidence Base

The framework proposed here is clinical and integrative, and its limits are defined by the evidence on which it relies. As summarized in Table 1, available adult esthetic studies establish efficacy and long-term acceptability but do not define the point at which repeated treatment may become less compatible in morphological or expressive terms [1,2,3,4,26,27]. Wrinkle scales do not distinguish between preserved compatibility and benefit maintained on a gradually adapted substrate [28,29,30]. The biological plausibility of muscle adaptation is supported by the literature on atrophy and remodeling, but the clinical relevance threshold in routine upper-face esthetics remains unspecified [5,6,7,8,31].

The specific limits of the present proposal are different in nature. The manuscript does not establish prevalence, thresholds, causality, or inter-rater reproducibility for any of the domains described. It does not operationalize the warning signs it proposes and does not define the conditions under which structural tolerance should be considered clinically compromised. Its contribution is to separate a clinically plausible compatibility problem from adjacent endpoints, to name its components, and to provide a provisional schema that future longitudinal studies can test, refine, or discard.

### 6.3. Research Priorities

Several research priorities follow from this framework. First, repeated-cycle longitudinal studies are needed to move beyond the single-cycle outcome logic. These studies should include serial documentation of standardized facial tasks and should not rely exclusively on patient satisfaction as the dominant endpoint [1,2,10,14,23]. Second, future designs should integrate wrinkle results, expressive results, and morphologic markers within the same protocol, rather than leaving these domains in a separate literature. Third, imaging-informed studies, including ultrasound-based approaches or other morphological assessment tools, can help clarify when the visible esthetic benefit remains aligned with the morphological preservation and when the two begin to diverge [8,9,18,19,20,24,32].

Fourth, more explicit modeling of dose, interval, regional pattern, and cumulative exposure is needed, since compatibility is unlikely to depend only on dose [5,6,7,26].

Within this fourth priority, BoNT-A formulation is itself a relevant variable. Currently used preparations—onabotulinumtoxinA, abobotulinumtoxinA, incobotulinumtoxinA, prabotulinumtoxinA, daxibotulinumtoxinA, and recently introduced ready-to-use relabotulinumtoxinA [33]—differ in complexing-protein content, diffusion field, onset, peak, and apparent clinical duration. In the perspective of structural tolerance, three formulation-dependent variables are likely to matter: (i) the spatial footprint of denervation per unit dose, which conditions which neighboring synergists are partially recruited into adaptation; (ii) the duration of effective denervation, which determines how long the muscle remains functionally unloaded between cycles and therefore the magnitude of the remodeling signal; (iii) the interval to retreatment permitted by the patient’s perceived return of motion, which may differ between formulations and shape cumulative exposure even when per-cycle dose is held constant. A preparation with a longer apparent duration may, in principle, reduce the per-cycle injection burden while also lengthening the cumulative denervation window per unit of calendar time. The structural-tolerance lens does not, in its current formulation, privilege any preparation; it predicts that the formulation, dose, and interval should be jointly modeled when compatibility trajectories are empirically studied [28,29,30,32,33]. Finally, future work should include observer-layered outcomes, including the perspectives of the clinician, patient, and third parties, because subtle changes in expression, naturalness, or facial readability may not be captured equally by evaluators [10,11,14,23,27,33,34].

Together, these priorities shift the focus from whether BoNT-A works for upper-face wrinkles to how broader longitudinal compatibility should be assessed and how early divergence might be identified more reliably. An illustrative mapping of candidate outcome domains for future empirical studies is provided in Supplementary Table S3, and a complementary mapping of candidate mechanistic and formulation-dependent variables is provided in Supplementary Table S4.

## 7. Conclusions

In routine upper-face esthetic BoNT-A, the clinical question extends beyond wrinkle reduction to whether repeated treatment remains acceptable over time in morphological, dynamic, expressive, and longitudinal terms. This paper introduces structural tolerance as a provisional clinical lens for that broader assessment problem. Organizing the issue into morphologic, dynamic–expressive, and longitudinal domains, and approaching it through a tiered assessment model, helps move follow-up beyond wrinkle response alone. Whether current outcome frameworks are sufficient to fully judge repeat treatment compatibility remains uncertain. Clarifying this gap is necessary for better longitudinal assessment and more rigorous study. The immediate practical direction is not to replace wrinkle-centered follow-up, but to extend it: serial documentation of regional form and facial dynamics, integrated with wrinkle outcome assessment, is feasible at every level of practice and does not require awaiting formal construct validation to begin. This is not a paper about how to inject; it is a paper about how to look more carefully at what injection produces over time.

## Figures and Tables

**Table 1:** What the current literature captures well and what remains underdefined in repeated upper-face esthetic BoNT-A.

Literature Domain	What It Supports	What It Does Not Establish	Why Current Assessment May Remain Incomplete
Wrinkle efficacy literature	BoNT-A is effective in reducing dynamic upper-face rhytides and remains a valid esthetic treatment for routine use.	It does not establish whether continued wrinkle improvement is accompanied by preserved regional form, expressive range, or broader longitudinal compatibility.	Wrinkle reduction alone cannot determine whether repeated treatment remains acceptable across morphologic and expressive dimensions.
Long-term adult esthetic studies	Repeated treatment may remain effective and satisfactory over time in many patients, without obvious treatment failure or gross loss of utility [1,2].	They do not fully define the boundary at which repeated treatment may become less natural, less expressively compatible, or less structurally coherent over time.	Durable efficacy and patient acceptance are important, but incomplete, proxies for long-term compatibility.
Atrophy/remodeling literature	Repeated chemodenervation is not biologically neutral and may be associated with muscle adaptation, atrophy, altered architecture, or incomplete recovery in some contexts [5,6,7,8].	It does not by itself establish when these changes become clinically relevant in routine upper-face esthetic practice, or how they should be assessed in everyday care.	Biological plausibility alone does not provide an operational framework for judging compatibility over repeated cycles.
Expressive/ psychological literature	The upper face contributes to emotional signaling, interpersonal readability, and socially meaningful facial modulation [10,11,14].	It does not define how wrinkle benefit, expressive preservation, and regional structural coherence should be integrated in follow-up.	A broader construct remains useful because esthetic success may coexist with subtler losses in expressive function or naturalness.
Pediatric non-esthetic literature	In non-esthetic settings and growing muscles, repeated BoNT-A exposure may be associated with persistent or slowly recovering changes in muscle morphology and growth trajectories [16,18,19].	These data cannot be directly extrapolated to routine adult upper-face esthetic treatment and do not prove equivalent facial outcomes.	They justify translational caution against assuming complete long-term biological neutrality and strengthen the rationale for a compatibility-oriented framework.

Legend: The table maps five literature domains against what each supports, what it does not establish, and why current assessment of repeated upper-face BoNT-A may remain incomplete when each domain is considered in isolation. BoNT-A = botulinum toxin type A.

**Table 2:** Hypothesized directional links between repeated BoNT-A exposure, the three tolerance domains, and the visible outcome captured by conventional endpoints. The table operationalizes, in tabular form, the structural-tolerance lens proposed in the preceding section: it is hypothesis-generating and is not intended as a validated causal model.

Hypothesized Link	Proposed Mechanism/Pathway	Role Within the Structural-Tolerance Window
Input → Morphologic tolerance Repeated BoNT-A exposure (dose, interval, cumulative)	Presynaptic chemodenervation followed by active synaptic and architectural recovery; over repeated cycles, fiber-type-selective atrophy and altered muscle architecture in the treated compartment.	Defines the regional substrate on which the next treatment cycle acts; not captured by wrinkle scales.
Morphologic→ Dynamic–expressive tolerance	Altered muscle architecture and reduced contractile reserve condition the available range of movement and the modulation of the upper face across tasks.	Explains how preserved wrinkle reduction can coexist with a narrowed expressive range and reduced task modulation.
Dynamic–expressive→ Longitudinal tolerance	Cumulative narrowing of expressive range and progressive loss of task-dependent variation across treatment cycles.	Generates a trajectory rather than a snapshot effect; visible only with serial comparison.
Longitudinal→Morphologic tolerance feedback	Cumulative exposure feeds back into regional form through reduced mechanical conditioning of the muscle–dermis interface and possible paracrine/secretome influence on adjacent soft tissue.	Closes the loop and provides a candidate biological route for progressive structural drift across cycles.
Dynamic–expressive tolerance→Visible outcome Wrinkle benefit + patient satisfaction	Standard wrinkle severity scales and patient-reported satisfaction measures preferentially capture the dynamic–expressive component of the treated cycle.	Captures only one slice of the cycle; may under-represent the longitudinal feedback loop and the cumulative regional cost.

Legend: Arrows (→) denote hypothesized directional links, not demonstrated causal pathways. The first row represents the upstream input to the structural-tolerance window; the last row represents the downstream slice captured by conventional endpoints. The three central rows describe the cross-domain interactions internal to the structural-tolerance lens. The feedback row (longitudinal→morphologic) closes the loop and is the principal reason a single-cycle outcome logic is considered insufficient. None of the listed mechanisms have been demonstrated specifically in the adult upper-face esthetic context.

**Table 3:** Domains relevant to longitudinal compatibility and candidate warning signs.

Domain	Focus	Candidate Warning Signs	What Wrinkle-Only Follow-Up May Miss
Morphologic tolerance	Regional form, contour harmony, and brow–periocular coherence across cycles	Periocular hollowing tendency; excessive brow flattening (relative to baseline); upper-face over-deflation; skeletonization cues	Progressive change in volume impression or brow–periocular balance despite maintained wrinkle reduction
Dynamic–expressive tolerance	Usable movement, task modulation, and expressive compatibility	Reduced expressive range; excessive brow immobility (relative to baseline); loss of task modulation; reduced periocular smile participation; overtreated appearance	Loss of functional movement or social readability despite satisfactory static outcome
Longitudinal tolerance	Stability of the wrinkle–naturalness–expressivity balance across repeated cycles	Benefit–naturalness decoupling; preserved satisfaction but declining expressive quality; narrowing treatment window; increasing need for caution; increasing reliance on protocol adjustment	A compatibility drift visible only with serial comparison, even when wrinkle benefit remains satisfactory

Legend: The three domains are analytically distinct but clinically interconnected. Warning signs are candidate indicators rather than validated diagnostic criteria.

**Table 4:** Illustrative tiered scaffold for approaching longitudinal compatibility: from routine clinical proxies to research-level compatibility markers.

Level/Setting	Core Elements	What It Supports	What It Cannot Support
Level 1/Routine injector	Standardized photographs and/or short videos in repose and during basic tasks; attention to brow balance, naturalness, dynamic range, and visible warning signs	Early detection of reduced tolerance and a basic judgment of whether wrinkle benefit remains compatible with an acceptable clinical result	Causality, tissue-level mechanisms, precise morphologic change, or formal boundary definition
Level 2/Structured clinician or training center	Serial cycle comparison; structured scoring; third-observer input when feasible; patient-reported naturalness or expressive comfort; optional pragmatic ultrasound	Stronger longitudinal judgment and better detection of emerging dynamic–expressive change	Definitive biologic mechanism, full generalizability, or research-grade quantification of structural change
Level 3/Research setting	Repeated-cycle longitudinal design; better-defined exposure patterns; integrated wrinkle, expressive, and morphologic endpoints; observer-layered outcomes; imaging-informed assessment	Empirical study of the compatibility boundary and of how benefit aligns or diverges from expressive and morphologic preservation across time	Universal thresholds or immediate transferability to every routine esthetic setting

Legend: The tiered model is illustrative rather than prescriptive. Each level defines what the assessment approach can and cannot support.

**Table 5:** Key clinical questions and considered responses regarding long-term upper-face esthetic BoNT-A.

Question	Considered Response
Does repeated BoNT-A injection cause muscle atrophy?	Evidence from imaging and histological studies suggests atrophy in multiple injection contexts, including a facial muscle (procerus) after a single dose [5,7,8,9]. Whether this occurs consistently in routine upper-face esthetic practice at standard doses and intervals, and whether it reaches clinical relevance, remains incompletely characterized. The evidence is sufficient to establish biological plausibility; it is not sufficient to establish clinical equivalence across all settings.
Can we assume that repeated upper-face chemodenervation is biologically neutral over time?	No. The available evidence does not support this assumption [5,6,7,8,9]. Imaging, neurophysiological, and histological studies document treatment-related muscle changes in multiple contexts. The magnitude and clinical relevance of these changes in routine adult esthetic practice are not fully established, but their existence is sufficient to argue against assuming complete biological neutrality across repeated cycles.
If wrinkle improvement is maintained across many treatment cycles, does that mean treatment is compatible?	Not necessarily. Wrinkle improvement and broader treatment compatibility are related but distinct [1,2,5,6]. Benefit can be maintained on a progressively adapted substrate without reflecting preserved regional form, expressive range, or longitudinal naturalness. Current wrinkle outcome measures are not designed to distinguish between these possibilities.
Can patient satisfaction be used as a proxy for long-term treatment compatibility?	Partly, but incompletely [10,11,14,23]. Satisfaction captures important dimensions of outcome but may not detect gradual narrowing of expressive range, subtle morphologic drift, or compatibility changes that are more visible to third-party observers or through serial comparison than to the patient. Observer-layered and dynamic–expressive assessments capture dimensions that satisfaction measures alone cannot. It should be noted that the link between patient satisfaction and failure to detect morphologic drift is inferential rather than directly demonstrated: no study has yet shown that satisfied patients systematically miss clinically relevant compatibility changes. The argument rests on the known limits of satisfaction as an outcome measure and on the construct-level gap between subjective acceptability and broader regional compatibility.
Can repeated upper-face BoNT-A reduce facial expressivity in ways not captured by standard assessment?	Yes, within the limits of available evidence [10,11,12,14,15]. Studies using observer-based rating, deep learning analysis, and structured emotional attribution tasks have documented that upper-face BoNT-A treatment affects how facial expressions are perceived and attributed by third parties, independently of patient satisfaction or wrinkle outcome. Standard wrinkle scales and satisfaction measures are not designed to detect these changes.
Should clinicians change their practice based on the concerns raised in this paper?	Not their injection practice, but yes their assessment practice. The framework proposed here does not call for dose reduction, interval extension, or treatment limitation. It does argue that serial documentation of regional form and facial dynamics—alongside wrinkle outcome—should become a routine part of long-term follow-up. This is a low-barrier step: standardized photography and short videos during basic facial tasks are already available in most clinical settings. The argument for doing so does not depend on formal construct validation; it depends only on the recognition that wrinkle response alone is an incomplete basis for judging repeated treatment over time.
Is “structural tolerance” a validated clinical construct?	No. The term is introduced here as a provisional clinical lens, not a validated endpoint [5,10,14]. It names a gap in current assessment frameworks and organizes candidate components for future empirical study. Validation would require longitudinal designs with integrated outcome measures, observer-layered assessment, and defined boundary conditions—none of which are yet available. The construct is offered as a problem formulation, not a clinical standard.

Note: Responses reflect the current state of evidence and are intended to make explicit what can and cannot be concluded at this stage. They do not constitute clinical recommendations or validated thresholds.

## Data Availability

No new data were created or analyzed in this study. Data sharing is not applicable to this article.

## Supplementary Materials

The following supporting information can be downloaded at: https://www.mdpi.com/article/10.3390/toxins18050232/s1, Supplementary Table S1: Expanded candidate warning signs for upper-face longitudinal compatibility; Supplementary Table S2: Illustrative assessment components by tier; Supplementary Table S3: Candidate outcome domains for future empirical studies of longitudinal compatibility. Supplementary Table S4: Candidate mechanistic and formulation-dependent variables relevant to longitudinal structural tolerance.
