# Supplementary material for: Beyond Wrinkle Efficacy: Toward a Broader Assessment of Longitudinal Compatibility in Routine Upper-Face Aesthetic BoNT-A
Source: Toxins (Basel). 2026 May 19;18(5):232. doi: 10.3390/toxins18050232 (PMC13211441; doi:10.3390/toxins18050232)
Supplement: Supplementary file 1 [file toxins-18-00232-s001.zip › toxins-4242802 - Supplementary_tables.pdf]

Supplementary Materials

Beyond Wrinkle Efficacy: Toward a Broader Assessment of
Longitudinal Compatibility in Routine Upper-Face Aesthetic BoNT-A

Supplementary Tables

This supplement contains three tables that expand on the candidate warning signs, assessment components, and outcome domains described in the main text. These tables are illustrative rather than prescriptive and do not constitute clinical guidelines or validated instruments.

Table S1: Expanded candidate warning signs for upper-face longitudinal compatibility.

Domain	Cue	Possible reading	Confounders / notes
Morphologic tolerance	Periocular hollowing tendency	Possible loss of regional fullness or altered brow–periocular balance across cycles	Check baseline anatomy, age-related volume change, weight change, lighting, and photo consistency
	Excessive brow flattening	Reduced upper-face contour dynamics or visually depleted brow support	Interpret against baseline brow shape, sex-related pattern, and frontalis recruitment
	Upper-face over-deflation appearance	Regional visual depletion not explained by line reduction alone	May overlap with generalized aging or concurrent soft-tissue change
	Regional skeletonization cues	Progressive prominence of bony landmarks or reduced soft-tissue buffering	Serial comparison is more informative than isolated cross-sectional viewing
Dynamic-expressive tolerance	Reduced expressive range	Movement becomes narrower than needed for wrinkle control	Best judged across tasks rather than in repose only
	Excessive brow immobility	Over-restriction of upper-face movement with possible expressive cost	Can coexist with high satisfaction and good wrinkle response
	Loss of modulation across tasks	Reduced ability to vary facial activation by context	Requires standardized brow elevation, frown, and smile documentation
	Diminished periocular smile participation	Weaker integration of the periocular region into smiling	Especially relevant when lateral orbicularis activity is treated
	Observer-rated overtreated appearance	Third-party impression of restricted or unnatural movement	Structured comparison helps reduce single-rater bias
Longitudinal tolerance	Decoupling between wrinkle benefit and naturalness	Continued line improvement with declining natural appearance	Usually requires serial documentation rather than a single visit
	Preserved satisfaction but declining expressive quality	Patient remains satisfied despite reduced dynamic-expressive compatibility	Reinforces that satisfaction alone is insufficient
	Progressive narrowing of the acceptable treatment window	Smaller margin between effective treatment and visible flatness or rigidity	May reflect cumulative exposure, evolving response, or regional adaptation
	Increasing need for caution across cycles	Greater technical difficulty in maintaining balanced outcomes	Interpret against dose, interval, and changes in treatment pattern
	Increasing reliance on protocol adjustment	Stable visible benefit requires shorter intervals, more touch-up, or less dose flexibility	Interpret against evolving treatment goals, technique changes, and cumulative exposure

Note: These cues are candidate observational prompts rather than diagnostic criteria or validated indicators of reduced compatibility. Each cue is potentially confounded and is best interpreted serially, contextually, and in relation to the broader clinical pattern.

Table S2: Illustrative assessment components by tier.

Level	Component	Role in assessment
Level 1	Standardized repose photographs	Minimum visual anchor for baseline and follow-up comparison of regional form
	Short video during brow elevation	Helps detect reduced frontalis amplitude not visible in static images
	Short video during frown	Supports assessment of glabellar activation and residual modulation
	Smile documentation when periocular area is treated	Helps judge periocular participation within the broader upper-face display
	Global judgment of naturalness, brow balance, and serial protocol stability	Screening-level recognition of possible reduced compatibility, including whether the result remains stable without increasing corrective adjustment; not formal scoring
Level 2	Serial comparison across cycles	Strengthens longitudinal judgment beyond isolated before/after review and helps detect growing reliance on protocol adjustment
	Third-observer evaluation	Reduces single-clinician interpretive bias when change is subtle
	Patient-reported naturalness / self-recognition / expressive comfort	Captures patient-perceived compatibility beyond satisfaction alone
	Structured expressive-preservation scoring	Makes dynamic-expressive follow-up more explicit and more comparable over time
	Optional simple ultrasound or pragmatic adjuncts	Adds morphologic support in selected cases without making imaging mandatory
Level 3	Repeated-cycle longitudinal design	Best suited to study compatibility trajectories rather than single responses
	Integrated endpoints including wrinkle, expressive, and morphologic assessment	Helps avoid separation of the three domains across unrelated studies
	Observer-layered outcome collection	Allows comparison of patient, clinician, and third-party perspectives
	Imaging-informed morphologic assessment	Helps study visible benefit versus morphologic adaptation over time
	Dose / interval / cumulative exposure modeling	Clarifies boundary conditions of repeated-cycle compatibility

Note: This table is intended as a feasibility scaffold rather than a guideline, standard of care, or validated assessment protocol.

Table S3: Candidate outcome domains for future empirical studies of longitudinal compatibility.

Outcome domain	Candidate variables	Why include it
Wrinkle outcome	Validated upper-face wrinkle scales; clinician-rated line severity; patient-rated wrinkle improvement	Necessary anchor for treatment benefit, but insufficient on its own
Morphologic compatibility	Brow contour harmony; periocular hollowing tendency; over-deflation cues; serial regional form comparison	Captures morphologic coherence beyond line reduction
Dynamic-expressive compatibility	Expressive range; brow mobility; task modulation; periocular smile participation; structured expressive-preservation scores	Captures whether treatment remains usable and socially readable
Longitudinal compatibility	Stability of naturalness across cycles; treatment-window width; need for progressive adjustment; shorter tolerated intervals; touch-up or corrective-refinement need; serial warning-sign accumulation	Captures trajectory rather than snapshot
Patient-reported compatibility	Naturalness; self-recognition; expressive comfort; longer-term acceptability	Captures dimensions not reducible to satisfaction alone
Observer-layered outcomes	Treating clinician rating; third-observer rating; blinded assessor comparison	Helps detect discordance between perspectives
Morphologic / imaging-informed markers	Ultrasound-derived thickness; morphology tracking; exploratory soft-tissue measures	Supports study of alignment or misalignment between visible outcome and morphologic change
Exposure-pattern variables	Dose per region; interval between treatments; cumulative dose; retreatment timing; regional injection pattern; touch-up frequency; protocol intensification over time	Needed to model compatibility boundary conditions
Contextual modifiers	Age; sex; baseline anatomy; treatment history; recruitment pattern; treated upper-face subregion	Important for stratification and interpretation of heterogeneity

Note: These domains are proposed for hypothesis-generating longitudinal studies and do not imply that longitudinal compatibility has already been established as a validated measurable endpoint.

Table S4: Candidate mechanistic and formulation-dependent variables relevant to longitudinal structural tolerance.

Level	Hypothesized mechanism / variable	Why it may matter for structural tolerance
Muscle fibre level	Fibre-type-selective atrophy (preferential type II involvement); reduced cross-sectional area; fibro-adipose substitution	Conditions the regional “substrate” on which the next treatment cycle acts; not captured by wrinkle scales
Muscle–dermis interface	Reduced mechanical conditioning of the overlying soft-tissue envelope secondary to sustained reduction of contractile activity	Plausible contributor to morphologic cues (periocular hollowing, brow flattening, regional skeletonization)
Paracrine / secretome	Altered myokine output of denervated/under-loaded muscle (myostatin, IL-6, IGF-1 axes); plausible influence on adjacent dermal fibroblast and adipocyte behaviour	Provides a candidate biological route linking muscle adaptation to peri-muscular soft-tissue remodeling
Formulation: spatial footprint	Diffusion / spread field per unit dose; complexing-protein content; delivery vehicle (lyophilized vs ready-to-use liquid)	Conditions which synergists are partially recruited into adaptation across cycles
Formulation: temporal footprint	Onset, peak, and apparent clinical duration of denervation; rate of axonal sprouting and synaptic recovery	Determines duration of functional unloading between cycles and therefore magnitude of remodeling signal
Formulation: re-treatment behaviour	Patient-perceived return of motion; interval-to-retreatment; cumulative exposure per unit of calendar time	A longer-duration preparation may reduce per-cycle burden while lengthening cumulative denervation window

Note: The mechanisms and variables in this table are hypothesis-generating. None has been demonstrated specifically in the adult upper-face aesthetic context; the table is intended to organize candidate routes for future imaging-, histology-, biomarker-, and exposure-modeling work.
